# Characterizing TDP‐43 involvement in vascular dementia

**DOI:** 10.1002/alz.71196

**Published:** 2026-02-14

**Authors:** Marconi Fung, Yuek Ling Chai, Yi‐Lin Cheng, Nishat Tabassum, Vernise J. T. Lim, Raj N. Kalaria, Dong‐Gyu Jo, Christopher P. Chen, Mitchell K. P. Lai, Thiruma V. Arumugam

**Affiliations:** ^1^ Department of Microbiology Anatomy Physiology and Pharmacology School of Agriculture Biomedicine and Environment La Trobe University Melbourne Australia; ^2^ La Trobe Institute for Molecular Science La Trobe University Melbourne Australia; ^3^ Department of Pharmacology Yong Loo Lin School of Medicine National University of Singapore Singapore Singapore; ^4^ Memory Aging and Cognition Centre National University Health System Singapore Singapore; ^5^ Translational and Clinical Research Institute Newcastle University Newcastle Upon Tyne United Kingdom; ^6^ School of Pharmacy Sungkyunkwan University Suwon Republic of Korea

**Keywords:** bilateral common carotid artery stenosis, chronic cerebral hypoperfusion, TDP‐43, vascular cognitive impairment, vascular dementia

## Abstract

**INTRODUCTION:**

Vascular dementia (VaD) is a major therapeutic challenge. Tar DNA‐binding protein 43 (TDP‐43), known for its role in neurodegeneration, may contribute to VaD pathogenesis under chronic cerebral hypoperfusion (CCH). This study investigates TDP‐43 dysregulation in VaD.

**METHODS:**

TDP‐43 and phosphorylated TDP‐43 (pTDP‐43) expression and localization were assessed in a VaD animal model, neuronal cells exposed to oxygen–glucose deprivation (OGD), and *post mortem* human brain tissues.

**RESULTS:**

Bilateral Common Carotid Artery Stenosis (BCAS)‐induced CCH led to increased pTDP‐43 and aberrant redistribution of both TDP‐43 and pTDP‐43. In vitro OGD triggered similar mislocalization. *Post mortem* VaD brains showed no TDP‐43 abnormalities, while Alzheimer's and mixed dementia cases exhibited marked pathology.

**DISCUSSION:**

TDP‐43 dysregulation appears early in VaD under hypoperfusive stress, distinguishing it from other dementia subtypes. These findings indicate that TDP‑43 may warrant further investigation as a potential early molecular feature of VaD.

**Highlights:**

- Tar DNA‐binding protein 43 (TDP‐43) is dysregulated early in vascular dementia models.
- Hypoperfusion triggers TDP‐43 mislocalization and phosphorylation.
- TDP‐43 pathology is absent in late‐stage human vascular dementia.
- TDP‐43 is a transient, novel target for vascular cognitive impairment.

## 1 BACKGROUND

Vascular dementia (VaD) ranks as the second most common cause of dementia worldwide and is linked to the highest mortality rate among dementia subtypes.^1^ A key driver of VaD pathogenesis is chronic cerebral hypoperfusion (CCH), a state of persistently reduced blood flow to the brain stemming from cerebrovascular compromise.^2^ This condition initiates a pathological cascade leading to cerebrovascular diseases, neuronal loss, and progressive cognitive decline.^2^, ^3^ The clinical trajectory of VaD is frequently severe, owing to its strong association with cardiovascular diseases.^4^ Two major contributors are post‐stroke dementia (PSD),^5^ which can develop shortly after a major cerebrovascular event, and small vessel disease (SVD),^5^, ^6^ which involves the cumulative effect of microvascular injuries such as microinfarcts, microbleeds, lacunar infarcts, and white matter lesions (WMLs). These insults, often associated with hypertension, diabetes, and atherosclerosis, disrupt critical neural networks, underpinning the gradual deterioration of cognitive function termed vascular cognitive impairment (VCI).^5^, ^6^, ^7^, ^8^

A hallmark of VaD, CCH can diminish cerebral blood flow by as much as 40%, triggering hypoxia‐induced cellular stress. This includes oxidative damage, mitochondrial failure, and heightened neuroinflammation,^2^ which collectively impair blood–brain barrier integrity and promote WML formation.^3^ Recent evidence points to Tar DNA‐binding protein 43 (TDP‐43) as a potential mediator in this cascade.^9^, ^10^ While TDP‐43′s pathological role is well‐established in amyotrophic lateral sclerosis (ALS),^11^, ^12^ frontotemporal dementia,^13^ and Alzheimer's disease (AD),^14^ its involvement in VaD is poorly understood. In healthy neurons, TDP‐43 is crucial for synaptic function and stress response.^15^ Under pathological conditions, however, it undergoes detrimental modifications, including hyperphosphorylation, nuclear‐to‐cytoplasmic mislocalization, and aggregation that are common processes across neurodegenerative diseases.^16^ These aberrant forms of TDP‐43 lose their normal function and can acquire toxic properties, potentially exacerbating neuroinflammation. While TDP‐43 pathology is a well‐established feature of several neurodegenerative diseases, its potential role in the context of cerebrovascular injury remains largely unexplored. Given that VCI and TDP‐43 proteinopathies share common downstream mechanisms, we sought to investigate whether TDP‐43 could represent a novel mechanistic link in VaD.

## 2 MATERIALS AND METHODS

### 2.1 Animals

Male C57BL/6 mice were obtained from the Animal Resource Centre (Australia) and maintained at the La Trobe Animal Research and Teaching Facility (LARTF) under controlled environmental conditions, including a 12‐hour light/dark cycle. Animals were provided unrestricted access to standard chow and water and were routinely monitored throughout the study period to ensure welfare and consistency. The study was conducted exclusively in male mice to control for sex as a biological variable, with the acknowledgment that this limits the generalizability of the findings to both sexes. All in vivo procedures were reviewed and approved by the La Trobe University Animal Ethics Committee (Protocol AEC21012) and conducted in compliance with the Australian Code for the Care and Use of Animals for Scientific Purposes as well as the National Institutes of Health (NIH) Guide for the Care and Use of Laboratory Animals.

**RESEARCH IN CONTEXT**

1. **Systematic review**: We comprehensively reviewed the literature on Tar DNA‐binding protein 43 (TDP‐43) proteinopathy and vascular dementia (VaD) using PubMed. While TDP‐43′s role in amyotrphic lateral sclerosis (ALS), frontotemporal dementia (FTD), and Alzheimer's disease is well‐established, its involvement in cerebrovascular disease and VaD remains poorly defined.
2. **Interpretation**: Our findings demonstrate that chronic cerebral hypoperfusion, a key driver of VaD, induces rapid TDP‐43 phosphorylation and mislocalization. Crucially, the absence of this pathology in late‐stage human VaD tissues suggests a novel, transient role for TDP‐43 in early disease pathogenesis, distinguishing VaD from other neurodegenerative TDP‐43 proteinopathies.
3. **Future directions**: Key questions include whether TDP‑43 mislocalization represents a causal driver or a downstream consequence, and whether TDP‑43 can be validated as a biomarker for early vascular cognitive impairment. In addition, defining the upstream molecular pathways and glial responses will be essential for understanding the pathogenic cascade. Finally, future work should assess whether TDP‑43 pathology preferentially emerges in subcortical or hippocampal regions.

### 2.2 Bilateral common carotid artery stenosis mouse model

Six‐month‐old male C57BL/6 mice were randomly allocated into two experimental groups: bilateral common carotid artery stenosis (BCAS) or Sham. Animals in the BCAS group were anesthetized using isoflurane and subjected to a midline cervical incision to expose both common carotid arteries (CCAs). Each CCA was then constricted by wrapping a microcoil (Sawane Spring Co. Ltd) around the vessel to induce CCH. Sham‐operated mice underwent the same surgical exposure of the CCAs without microcoil placement. Animals were monitored postoperatively until fully recovered and able to access food and water freely. Euthanasia was performed via carbon dioxide inhalation at designated time points for downstream analyses.

### 2.3 Human tissue

Frozen tissue blocks from the prefrontal cortex (Brodmann area 9) of individuals neuropathologically diagnosed with control (*n* = 8), VaD (*n* = 8), mixed dementia (*n* = 8), AD (*n* = 8), post‐stroke non‐demented (PSND, *n* = 8), and PSD (*n* = 7) were obtained from the Newcastle Brain Tissue Resource, United Kingdom, were carefully thawed on ice. Meningeal layers and underlying white matter were removed by dissection to isolate gray matter regions.

### 2.4 Cell culture and in vitro model of CCH

SH‐SY5Y human neuroblastoma cells (ATCC CRL‐2266) were cultured in DMEM/F‐12 with GlutaMAX, 10% heat‐inactivated fetal bovine serum (FBS), and 1% penicillin‐streptomycin at 37°C, 5% CO_2_. Morphology was monitored via phase‐contrast microscopy. At ∼80% confluency, cells were passaged using trypsin, centrifuged, resuspended, and counted (CellDrop BF, Denovix) before seeding at 25,000 cells/coverslip in 12‐well plates. Primary cortical neurons were isolated from E16 C57BL/6J mouse cortices and cultured in Neurobasal Plus medium with 2% B‐27, 0.5 mM GlutaMAX, and 0.05% Gentamicin. Cultures were maintained for 7 days at 37°C; neuronal purity was ∼95%. Cells were exposed to normoxic or oxygen–glucose deprivation (OGD) conditions simulating CCH. Controls used standard media under 5% CO_2_. Specifically, oxygen levels in the incubator were reduced from 18% to 10.8%, and glucose concentration in the culture media was lowered from 0.315% w/v to 0.189% w/v (40% glucose reduction). SH‐SY5Y cells were treated for 6, 12, or 24 h; primary neurons for 6 or 24 h.

### 2.5 Immunoblot analysis

Human brain lysates (40 µg protein) were separated on 10% sodium dodecyl sulfate–polyacrylamide gel electrophoresis (SDS‐PAGE) gels (Mini‐PROTEAN, Bio‐Rad) and transferred to polyvinylidene fluoride (PVDF) membranes. Membranes were blocked in phosphate‐buffered saline with Tween 20 (PBST) with 5% blotting‐grade blocker (Bio‐Rad) for 1 hour at room temperature, then incubated overnight at 4°C with primary antibodies against TDP‐43, pTDP‐43 (serine 409/410), and β‐actin in PBST with 5% bovine serum albumin (BSA). Mouse cortex lysates (20 µg) were run on 4%–15% gradient SDS‐PAGE gels and transferred to nitrocellulose membranes. Blocking used 1% fish skin gelatin in Tris‐buffered saline with Tween 20 (TBST) for 2 hours. Membranes were incubated overnight at 4°C with the same primary antibodies. After three 10‐minute washes (PBST for human, TBST for mouse), horseradish peroxidase (HRP) ‐conjugated secondary antibodies (Jackson ImmunoResearch) were applied for 1 hour at room temperature. Bands were visualized using Luminata Forte (human) or enhanced chemiluminescence ECL (Bio‐Rad; mouse) and imaged with Alliance 4.7 (UVItec) or ChemiDoc (Bio‐Rad). Image Lab (v6.1) and ImageJ were used for processing and quantification.

### 2.6 Immunohistochemistry and immunocytochemistry

Paraffin‐embedded mouse brain sections (4 µm) were deparaffinized, rehydrated, and subjected to antigen retrieval (sodium citrate buffer, pH 6). Sections were blocked (3% horse serum, 0.3% Triton X‐100 in PBS), then incubated overnight at 4°C with anti‐TDP‐43 or anti‐pTDP‐43 antibodies (Affinity Biosciences). After PBS–Tween washes, Alexa Fluor 488 secondary antibody (Abcam) was applied for 1 hour at room temperature. NeuroTrace (Thermo Fisher) labeled neurons; slides were mounted with ProLong Diamond Antifade with 4′,6‐diamidino‐2‐phenylindole (DAPI). Imaging used ZEISS 780 confocal microscope; analysis was performed with Imaris (v9.5). For immunocytochemistry (ICC), cells were fixed (4% paraformaldehyde), permeabilized, blocked, and incubated overnight with primary antibodies (anti‐TDP‐43, anti‐pTDP‐43, anti‐MAP2). Alexa Fluor 488/594 secondary antibodies were applied for 1 hour in the dark. SH‐SY5Y cells received additional blocking before anti‐βIII‐tubulin incubation and Alexa Fluor 594 labeling. Final imaging used ZEISS 780/980 microscopes; data were processed with Imaris (v9.5).

### 2.7 Imaris image processing

Z‐stack confocal images of immunohistochemistry (IHC) and ICC preparations were acquired using a Zeiss LSM 780 or 980 confocal microscope. Image stacks were processed and rendered in three dimensions using Imaris software (version 9.5). Surface objects were generated for nuclear staining (DAPI), target proteins (TDP‐43 or phosphorylated TDP‐43), and cytoplasmic markers specific to each modality, NeuroTrace for IHC and βIII‐Tubulin or MAP2 for ICC. To assess cytoplasmic localization, masking was applied to merge the protein signal with its corresponding cytoplasmic marker, generating a new channel representing cytoplasmic TDP‐43 or pTDP‐43.

### 2.8 Quantification and statistical analysis

Timepoint, experimental group, and protein identity were blinded prior to image processing and gel quantification. Blinding was maintained throughout the workflow and only lifted after all measurements were completed to minimize observer bias. Mouse cortex immunoblot images were processed and band intensities quantified using ImageJ software. Statistical analyses were performed using GraphPad Prism. A two‐way analysis of variance (ANOVA) followed by Fisher's least significant difference (LSD) post‐hoc test was applied, with data presented as mean ± standard error of the mean (SEM). Statistical significance was defined as *p* < 0.05. IHC and ICC images were processed and quantified using Imaris software (version 9.5), with subsequent statistical analysis conducted in GraphPad Prism (version 10.2.3). A two‐way ANOVA with Fisher's LSD post‐hoc test was used, and data were reported as mean ± SEM, with *p* < 0.05 considered statistically significant. Human Western blots were processed and quantified with UVIBand software (UVItec) for quantification and statistical analysis was conducted using GraphPad Prism (v. 10.4.1). A one‐way ANOVA and Fisher's LSD post‐hoc test were performed, where data were presented as a ±SEM where a *p*‐value < 0.05 was considered statistically significant.

## 3 RESULTS

### 3.1 Chronic cerebral hypoperfusion induces cytoplasmic mislocalization and hyperphosphorylation of TDP‐43 in cortical and hippocampal neurons

**FIGURE 1:**
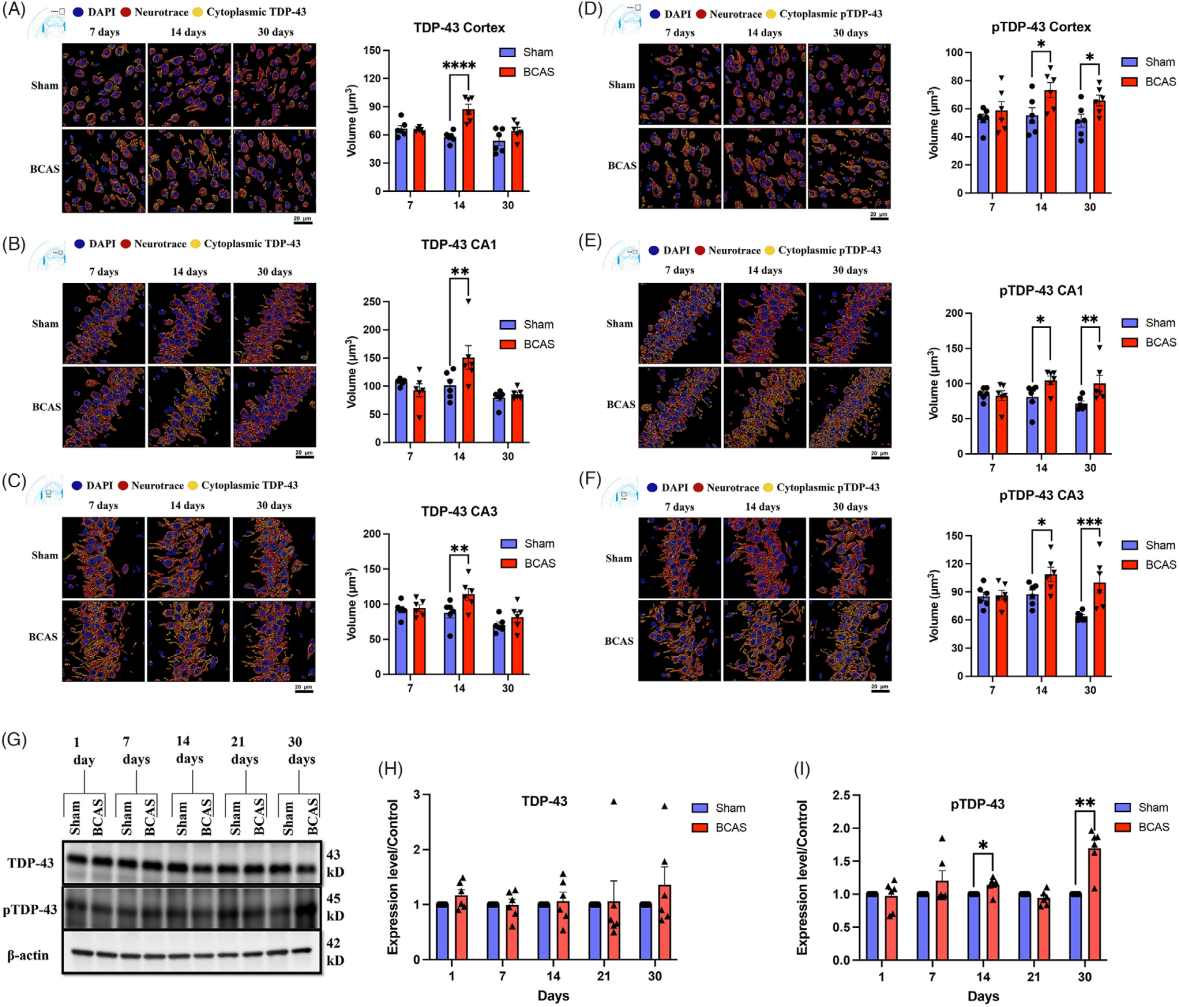
Chronic cerebral hypoperfusion (CCH) induces cytoplasmic mislocalization of Tar DNA‐binding protein 43 (TDP‐43) and phosphorylated TDP‐43 (pTDP‐43) in the cortex and hippocampus. Immunofluorescence staining was performed on 4 µm paraffin‐embedded brain sections. Sections were labeled with anti‐TDP‐43 or anti‐pTDP‐43 antibodies (green), NeuroTrace neuronal marker (red), and 4′,6‐diamidino‐2‐phenylindole (DAPI) nuclear stain (blue). Only cytoplasmic colocalization of TDP‐43 or pTDP‐43 with NeuroTrace in yellow is shown. Representative high‐magnification images (60×) demonstrate increased cytoplasmic localization of TDP‐43 and pTDP‐43 in neurons following CCH, compared to sham controls. (A–C) Representative images and quantification of cytoplasmic TDP‐43 expression in the cortex, CA1, and CA3 regions, respectively. (D–F) Corresponding data for pTDP‐43. Quantitative analysis was performed using two‐way analysis of variance (ANOVA), with data presented as mean ± standard error of the mean (SEM). Statistical significance was defined as *p* ≤ 0.05 (*), *p* ≤ 0.01 (**), *p* ≤ 0.001 (***), and *p* ≤ 0.0001 (****). Scale bar = 20 µm; *n* ≥ 3 mice per group. Western blot analysis was conducted on cortical tissue lysates from sham and bilateral common carotid artery stenosis (BCAS) mice (*n* = 6 per group), with β‐actin used as a loading control. (G) Representative blots for TDP‐43 and pTDP‐43. (H, I) Present quantification of TDP‐43 and pTDP‐43 protein levels, respectively. Statistical analysis was performed using two‐way ANOVA, with data expressed as mean ± SEM. Significance was defined as *p* ≤ 0.01 (**).

To assess the impact of CCH on TDP‐43 and phosphorylated form (pTDP‐43), we examined their expression levels and subcellular localization in brain tissues of mice subjected to BCAS compared to sham‐operated controls. Immunofluorescence staining was performed at early‐stage (7 and 14 days) and intermediate‐stage (30 days) post‐surgery to evaluate the spatial distribution of TDP‐43 and pTDP‐43 in the cortex and hippocampal subregions (CA1 and CA3). In parallel, immunoblot analysis was conducted on cortical lysates at 1, 7, 14, 21, and 30 days post‐surgery to quantify protein expression levels. Colocalization studies using Neurotrace (neuronal marker) and respective protein stains revealed a time‐dependent cytoplasmic redistribution of TDP‐43 and pTDP‐43 in BCAS mice. Specifically, TDP‐43 exhibited significant cytoplasmic accumulation at 14 days post‐BCAS in both cortical and hippocampal neurons (Figure 1A–C). pTDP‐43 mislocalization was more pronounced, with marked cytoplasmic presence observed at both 14 and 30 days post‐BCAS in the cortex and hippocampal CA1 and CA3 regions (Figure 1D–F), indicative of persistent aggregate formation. No significant changes in localization were detected at 7 days post‐surgery for either TDP‐43 or pTDP‐43.

Immunoblot analysis corroborated the IF findings. Total TDP‐43 levels remained unchanged across all time points in BCAS mice relative to sham controls (Figure 1G–I). In contrast, pTDP‐43 levels showed a significant upregulation at 14 and 30 days post‐BCAS, while no differences were observed at 1, 7, or 21 days (Figure 1H–I). These results suggest that CCH induces sustained hyperphosphorylation of TDP‐43, potentially contributing to its cytoplasmic mislocalization and aggregation.

### 3.2 OGD induces time‐dependent cytoplasmic mislocalization of TDP‐43 and pTDP‐43 in neuronal cells

**FIGURE 2:**
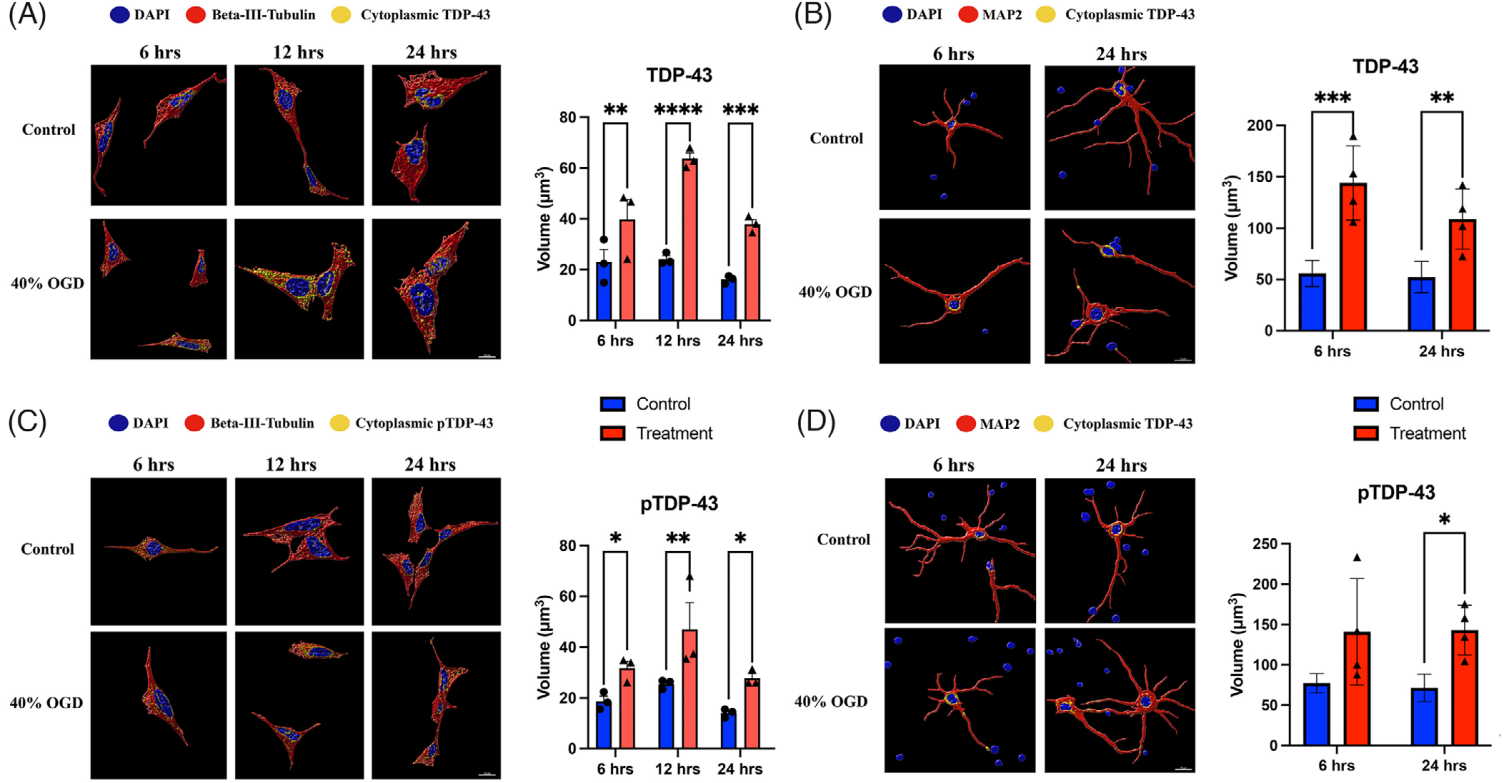
Cytoplasmic mislocalization of Tar DNA‐binding protein 43 (TDP‐43) and the phosphorylated form (pTDP‐43) following 40% oxygen–glucose deprivation (OGD). Immunocytochemistry was performed on SH‐SY5Y cells and primary cortical neurons (*n* = 3–4 biological replicates) to assess cytoplasmic redistribution of TDP‐43 and pTDP‐43 in response to 40% OGD. Cells were stained with anti–TDP‐43 or anti–pTDP‐43 (green), anti–βIII‐Tubulin or MAP2 (red), and 4′,6‐diamidino‐2‐phenylindole (DAPI) (blue). Only cytoplasmic colocalization of TDP‐43 or pTDP‐43 with neuronal markers is shown in yellow in merged images. Representative confocal micrographs were acquired at 60× magnification. (A) SH‐SY5Y and (B) primary neuron show cytoplasmic TDP‐43 localization and quantification. (C) SH‐SY5Y and (D) primary neuron show pTDP‐43. Quantitative analysis of cytoplasmic TDP‐43/pTDP‐43 volume (µm^3^) was performed using a two‐way analysis of variance (ANOVA). Data are presented as mean ± standard error of the mean (SEM). Statistical significance was defined as *p* ≤ 0.05 (*), ≤ 0.01 (**), ≤ 0.001 (***), and ≤ 0.0001 (****). Scale bars = 15 µm.

To further investigate the cellular dynamics of TDP‐43 and pTDP‐43 under CCH‐like conditions, we employed an in vitro OGD model using SH‐SY5Y neuroblastoma cells and primary cortical neurons. Cells were exposed to 40% OGD for 6, 12, and 24 hours, corresponding to early (6 hours), intermediate (12 hours), and late (24 hours) OGD exposure windows. ICC revealed a marked time‐dependent cytoplasmic redistribution of both TDP‐43 and pTDP‐43 following 40% OGD exposure (Figure 2A–D). In SH‐SY5Y cells, significant cytoplasmic accumulation of TDP‐43 was observed as early as 6 hours, with progressive intensification at 12 and 24 hours post‐OGD (Figure 2A). Similarly, pTDP‐43 showed pronounced cytoplasmic localization at 6, 12, and 24 hours, suggesting phosphorylation‐dependent mislocalization and potential aggregate formation (Figure 2C). Primary cortical neurons exhibited comparable patterns, with cytoplasmic TDP‐43 and pTDP‐43 accumulation evident at 6 and 24 hours post‐OGD (Figure 2B,D). Quantitative volumetric analysis confirmed significant increases in cytoplasmic TDP‐43 and pTDP‐43 under 40% OGD conditions compared to controls, reinforcing the hypothesis that metabolic stress drives aberrant subcellular trafficking of TDP‐43. These findings support the in vivo observations and suggest that OGD‐induced metabolic stress is sufficient to trigger early and sustained mislocalization of TDP‐43 and pTDP‐43 in neuronal cells, potentially contributing to neurodegenerative cascades associated with CCH.

### 3.3 TDP‐43 and pTDP‐43 expression remains unaltered in late‐stage vascular dementia compared to AD

**FIGURE 3:**
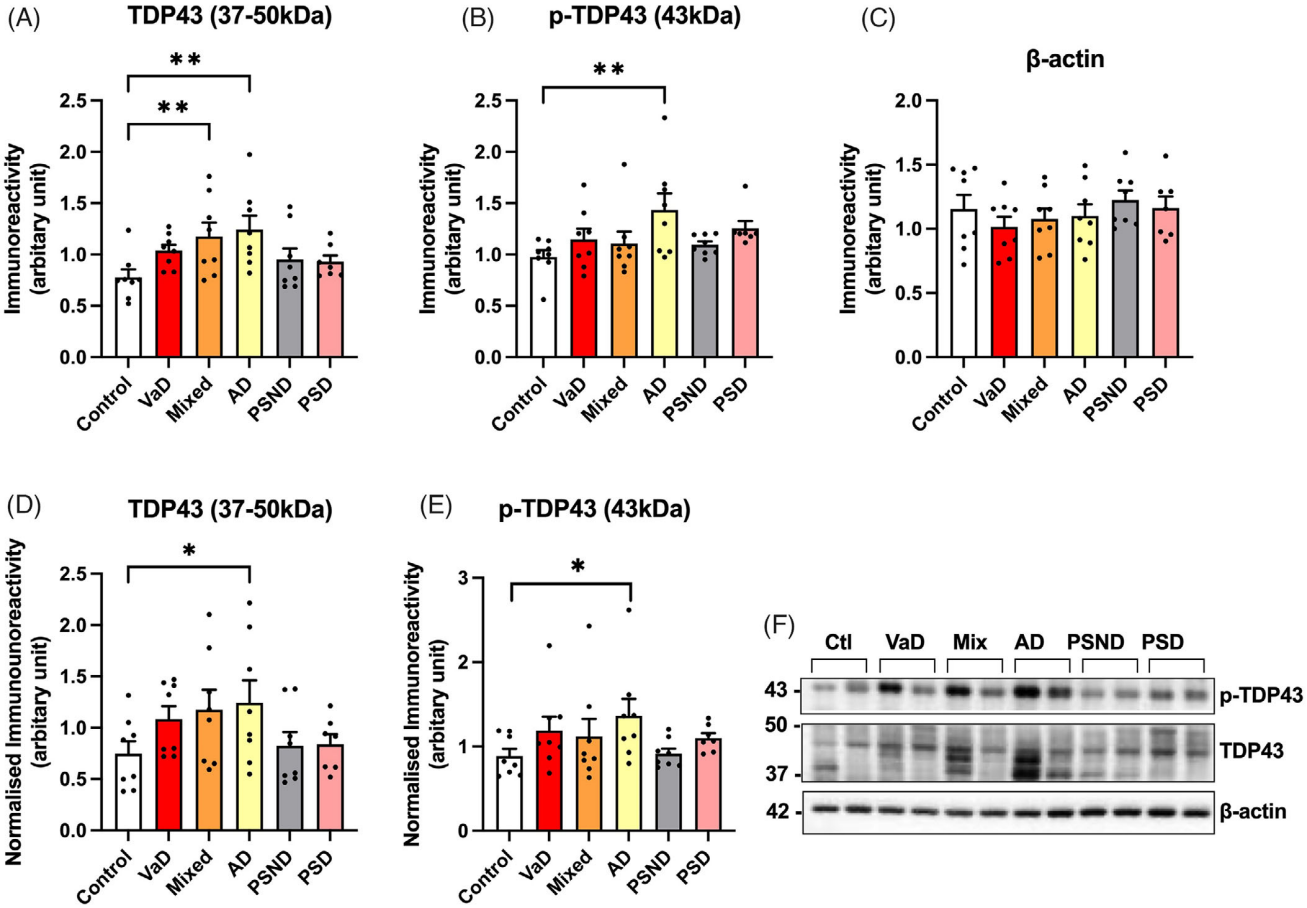
Altered expression of Tar DNA‐binding protein 43 (TDP‐43) and phosphorylated TDP‐43 (pTDP‐43) in vascular and related dementias. Western blot analysis was performed on *post mortem* brain tissue from Brodmann area 9 across six diagnostic groups: no disease control (Control, *n* = 8), vascular dementia (VaD, *n* = 8), mixed dementia (Mixed, *n* = 8), Alzheimer's disease (AD, *n* = 8), post‐stroke no dementia (PSND, *n* = 8), and post‐stroke dementia (PSD, *n* = 7). (A–C) Raw immunoreactivity levels for TDP‐43 (37–50 kDa), pTDP‐43 (43 kDa), and β‐actin, respectively. (D, E) Normalized TDP‐43 and pTDP‐43 levels relative to β‐actin. (F) Representative Western blot images for each protein across diagnostic groups. Statistical analysis was performed using one‐way analysis of variance (ANOVA). Data are presented as mean ± standard error of the mean (SEM). Significance thresholds were defined as *p* ≤ 0.05 (*), *p* ≤ 0.01 (**).

To evaluate whether alterations in total TDP‐43 and pTDP‐43 are implicated in the terminal neuropathological late stage of VaD, we performed immunoblot analysis on *post mortem* brain samples obtained from the Newcastle Brain Bank, United Kingdom. Samples included individuals diagnosed with AD, VaD, PSD, PSND, and mixed dementia, and age‐matched healthy controls. Quantitative analysis revealed a significant upregulation of both TDP‐43 and pTDP‐43 immunoreactivity in AD patients compared to controls (Figure 3A–F), consistent with previous reports linking TDP‐43 pathology to AD progression. In contrast, no significant changes in TDP‐43 or pTDP‐43 expression were observed in VaD, PSD, or PSND groups relative to controls, despite comparable β‐actin loading across all samples. The mixed dementia group had significantly elevated TDP‐43 compared to controls (Figure 3A). These findings suggest that, unlike in AD, dysregulation of TDP‐43 and its phosphorylation state may not be a prominent feature in the late‐stage pathology of VaD. This distinction may underscores the potential mechanistic divergence between neurodegenerative and vascular dementias and highlights the need for disease‐specific biomarkers and therapeutic targets.

## 4 DISCUSSION

This study demonstrates that CCH, a key feature of VaD, triggers pathological TDP‐43 changes, namely cytoplasmic mislocalisation and hyperphosphorylation, in both in vivo and in vitro models. In the BCAS mouse model, time‐dependent cytoplasmic accumulation of TDP‐43 and pTDP‐43 was observed in cortical and hippocampal neurons, with elevated pTDP‐43 despite stable total TDP‐43 levels, implicating phosphorylation in its aberrant redistribution. These results mirror hallmark features of TDP‐43 proteinopathies in neurodegenerative diseases, such as ALS and AD, and suggest that similar mechanisms may be triggered by vascular insults.^12^, ^13^, ^14^, ^15^, ^16^, ^17^ Our in vitro OGD model of CCH further validated these observations, demonstrating that metabolic stress alone is sufficient to induce cytoplasmic mislocalization of TDP‐43 and pTDP‐43 in neuronal cells. The rapid onset and persistence of these changes across time points indicates that CCH‐associated stressors, such as hypoxia, oxidative damage, and energy failure can disrupt TDP‐43 homeostasis. Importantly, the consistency between SH‐SY5Y cells and primary cortical neurons strengthens the translational relevance of these findings. However, future studies incorporating fractionation‑based approaches will be essential to more precisely define the subcellular dynamics of TDP‑43 under CCH conditions. The relatively short hypoperfusion window used in this model limits the extent to which long‑term or potentially transient TDP‑43 alterations can be inferred. Because human vascular cognitive impairment progresses over many years, extended time points, aged animals, and longitudinal sampling will be essential to determine whether TDP‑43 changes normalize, persist, or evolve toward insoluble species.

A notable observation from this study is the absence of persistent TDP‐43 or pTDP‐43 abnormalities in *post mortem* human VaD, despite clear early‐stage alterations in the BCAS model. This apparent discrepancy may reflect stage‐specific or reversible TDP‐43 responses to CCH. Experimental hypoperfusion models such as BCAS typically induce robust neuronal stress within 2–4 weeks. However, some molecular changes, including oxidative and inflammatory markers, normalize by 8–12 weeks once collateral perfusion is partially restored. Although our study assessed up to 30 days post‐BCAS, it remains possible that prolonged hypoperfusion (>60 days) results in either normalization of phosphorylated TDP‐43 or conversion to insoluble species undetectable by conventional immunoblotting.

The absence of significant TDP‐43 pathology in the VaD cortex could reflect several methodological and pathological factors. Human analysis in this study was confined to the prefrontal cortex (Brodmann area 9), whereas the BCAS model and prior imaging studies indicate that hippocampal and white‐matter regions sustain the most severe hypoperfusion. Consequently, disease‑relevant TDP‑43 mislocalization may be concentrated in these more vulnerable regions, which were not examined here. Future studies should incorporate targeted sampling from hippocampal and subcortical white matter, paired with subcellular fractionation, to delineate region‑ and compartment‑specific TDP‑43 dynamics. Finally, the interpretation of human *post mortem* data must account for comorbid pathologies. Although cases were rigorously classified using CERAD and NINDS‑AIREN criteria to separate pure AD from VaD, sub‑threshold mixed pathology or associated cardiovascular conditions may contribute to cohort variability. These inherent limitations of *post mortem* studies underscore the need for cautious interpretation and highlight the importance of integrating multimodal approaches in future research. Furthermore, the upstream molecular pathways that mechanistically link chronic hypoperfusion to altered TDP‑43 processing, however, remain to be defined. Elucidating the roles of specific kinases, phosphatases, nucleocytoplasmic transport mechanisms, and glial contributions will be essential to understand the broader pathogenic cascade and to assess whether TDP‑43–related alterations represent an early and actionable therapeutic target.

Taken together, our findings propose a model in which CCH initiates TDP‐43 mislocalization and phosphorylation, potentially contributing to neuronal dysfunction and cognitive decline. However, the absence of sustained TDP‐43 pathology in late‐stage human VaD underscores the complexity of its role and highlights the need for longitudinal studies and region‐specific analyses with increased sample size. It is also possible that TDP‐43 contributes to early vulnerability or acts synergistically with other molecular pathways, such as neuroinflammation or epigenetic dysregulation, which warrant further investigation.^18^ From a translational perspective, these results open new avenues for exploring TDP‐43 as a biomarker of early cerebrovascular stress and as a potential therapeutic target. Moreover, the divergence between AD and VaD in TDP‐43 pathology reinforces the need for dementia subtype‐specific diagnostic and therapeutic strategies.

## CONFLICT OF INTEREST STATEMENT

The authors report no competing interests. Author disclosures are available in the Supporting Information.

## CONSENT STATEMENT

The collection and study of brain tissues have received Institutional Review Board approval in both the UK (08/H1010/4) and Singapore institutions (NUS 12‐062E), and informed consent was obtained from patients’ next‐of‐kin or authorized caregivers prior to removal of brain.

## ETHICS STATEMENT

All in vivo experimental procedures were approved by La Trobe University (Ethics approval number: AEC21012) Animal Care and Use Committees and performed according to the guidelines set forth by the Australian Code for the Care and Use of Animals for Scientific Purposes (8th edition) and confirmed NIH Guide for the Care and Use of Laboratory Animals. *Post mortem* frozen human brain tissues from vascular dementia, mixed dementia, Alzheimer's disease, post‐stroke non‐demented, and post‐stroke dementia, and age‐matched normal control subjects were obtained from the Newcastle Brain Tissue Resource, Institute for Ageing and Health, Newcastle University.

## Supporting information



Supporting information
